# Recurrent Lelliottia amnigena Septic Arthritis in an Immunocompetent Child

**DOI:** 10.7759/cureus.116790

**Published:** 2026-09-23

**Authors:** Jesse Kooistra, Matthew Eisenhardt, John C Christenson, Jonathan A Mayhew

**Affiliations:** 1 Department of Pediatrics, Western Michigan University Homer Stryker M.D. School of Medicine, Kalamazoo, USA; 2 Department of Pediatric Infectious Diseases, Ryan White Center for Pediatric Infectious Diseases and Global Health, Indianapolis, USA

**Keywords:** enterobacter, gram-negative bacterial infection, lelliottia amnigena, pediatric case, septic arthritis of knee

## Abstract

Human infections secondary to *Lelliottia amnigena* are rare and have been reported almost exclusively in immunocompromised hosts or following penetrating trauma. We present the case of a previously healthy four-year-old girl who developed left knee septic arthritis caused by *L. amnigena* without a history of penetrating trauma. Her diagnosis was delayed by initially negative synovial fluid cultures, inconsistent organism nomenclature between laboratories, and a weakly positive *Borrelia burgdorferi* enzyme-linked immunosorbent assay (ELISA) that broadened the differential. Her symptoms resolved at the completion of her first treatment course. Approximately 18 months later, she re-presented with recurrent left knee infection due to *L. amnigena*. She was treated with surgical drainage followed by a three-month course of trimethoprim/sulfamethoxazole with complete resolution. This case highlights an unusual pathogen in pediatric septic arthritis and underscores the importance of considering extended or tailored antimicrobial therapy when managing rare organisms with poorly characterized virulence and relapse potential.

## Introduction

Septic arthritis is a medical emergency. In the pediatric population, the most commonly implicated pathogens are *Staphylococcus aureus*, *Streptococcus pyogenes*, and *Kingella kingae* [1,2]. Less frequently, gram-negative bacilli are identified, typically in the setting of risk factors such as immunodeficiency, indwelling prosthetic material, or penetrating trauma. *Lelliottia amnigena* (formerly *Enterobacter amnigenus*) is a gram-negative, facultatively anaerobic rod that is predominantly a plant-associated bacterium [3]. Human infection with *L. amnigena* is rare, with a small number of cases reported [4]; when it occurs, it is typically in the context of immunosuppression [5,6], invasive devices [5], or traumatic inoculation [7,8]. Its virulence factors and clinical spectrum are not well defined.

We report an unusual case of *L. amnigena* septic arthritis in a previously healthy pediatric patient who developed recurrent infection about 18 months later despite treatment of the initial infection. This case underscores the importance of considering uncommon organisms when evaluating septic arthritis, as well as the potential for recurrence despite apparent appropriate antimicrobial therapy.

This work was previously presented as a poster at the 43rd Annual Kalamazoo Community Medical and Health Sciences Research Day in April 2025 and the CMDA National Convention in May 2025.

## Case presentation

A four-year-old girl presented to the emergency department (day 0), with swelling, erythema, and pain of the left knee. Two days prior, the patient was running in the yard, tripped, and landed on the grass. There was no obvious injury or open wound at the time of the fall, and the patient had no symptoms until the day of presentation. She did not have fever, rash, or other joint involvement and never developed systemic symptoms during her illness course. The patient had no significant past medical, surgical, or family history. Physical examination revealed a warm, swollen, erythematous left knee with decreased range of motion secondary to pain (Figure 1). The remainder of the examination was unremarkable. An X-ray of the left knee demonstrated soft tissue swelling without a fracture (image not available), and a white blood cell count (WBC) showed neutrophil predominance without leukocytosis. The patient was started on oral cephalexin for a possible soft tissue infection for seven days.

**Figure 1:**
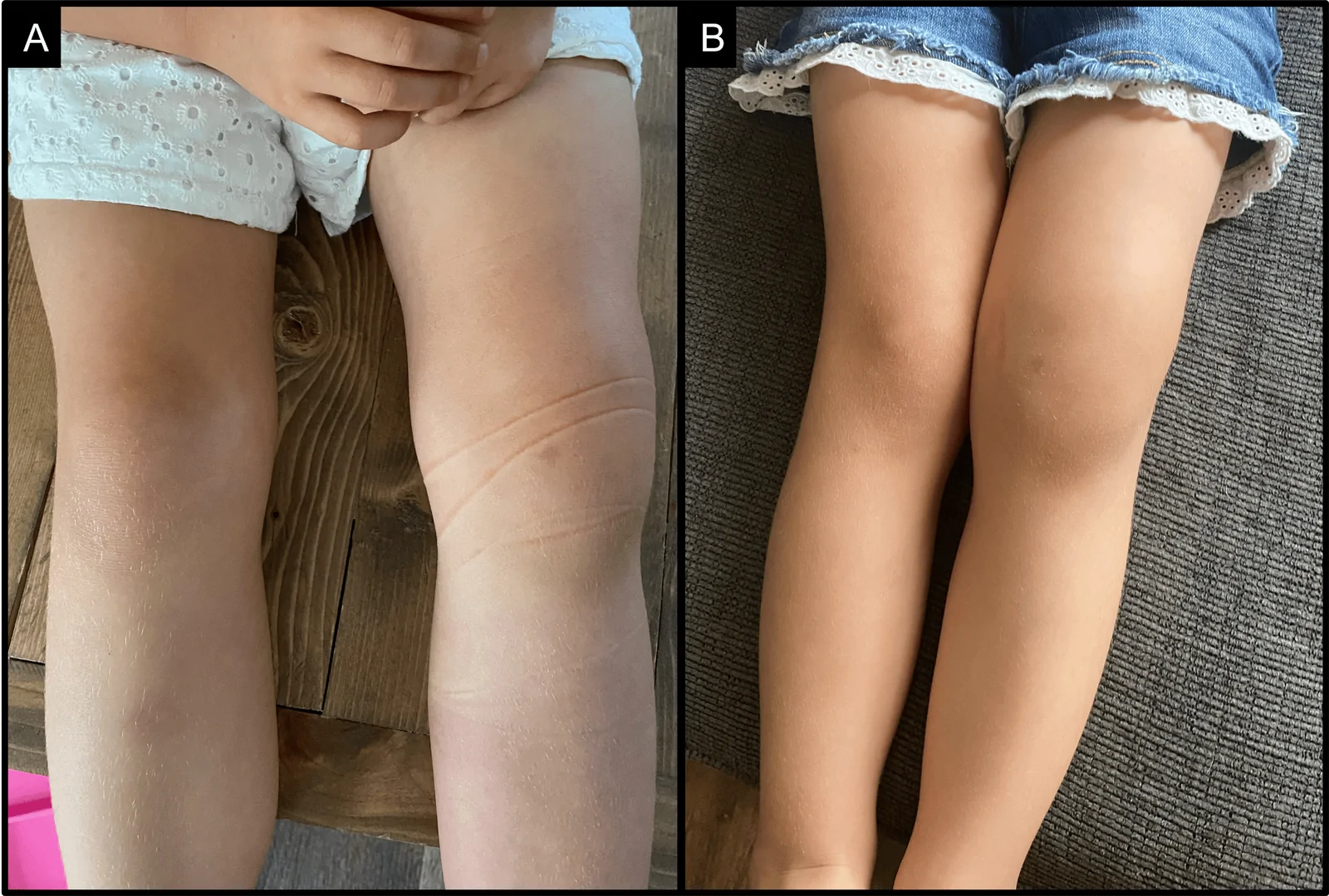
Left knee effusion during the patient's initial illness (A) Left knee effusion five days after an apparent minor injury. (B) The patient's effusion progressed and persisted until she had surgical intervention and treatment for confirmed *L. amnigena* septic arthritis.

Magnetic resonance imaging (MRI) of the knee was obtained on day 3 due to persistent symptoms and demonstrated a joint effusion with uniform synovitis and reactive lymph nodes in the popliteal space (image not available). Blood cultures collected at this time were negative. On day 9, aspiration of the left knee yielded cloudy synovial fluid with 42,300 WBC/µL (normal <200 WBC/µL) but negative cultures. On day 16, a repeat aspiration revealed 235,000 WBC/µL with >90% neutrophils, and cultures grew *Enterobacter* species reportedly susceptible to cefazolin. This could not be verified by the available documentation. This was performed at an outside hospital, and further speciation was not pursued. One of four blood culture bottles (two sets of aerobic and anaerobic cultures) was positive for *Enterobacter* (*Lelliottia*) *amnigena* by matrix-assisted laser desorption/ionization time-of-flight (MALDI-TOF). The connection between the positive blood and synovial fluid cultures was not ascertained at this time. She was continued on cephalexin based upon reported susceptibility profiles. Her symptoms persisted after two weeks of antibiotics despite adherence to, and completion of, the cephalexin course.

The differential diagnosis was broadened to transient synovitis, Lyme arthritis, trauma, or an autoimmune rheumatologic condition such as juvenile idiopathic arthritis. On day 51, the pediatric infectious diseases service was consulted which resulted in admission for suspected septic arthritis. She had a normal complete blood count and comprehensive metabolic panel, an elevated C-reactive protein (CRP) of 11.3 mg/dL (normal <1.0), and a Westergren erythrocyte sedimentation rate (ESR) of 57 mm/hr (normal <20). A repeat arthrocentesis showed 68,073 WBC/µL with 96% neutrophils. A *Borrelia burgdorferi* enzyme-linked immunosorbent assay (ELISA) was weakly positive, but the confirmatory Western blot was not consistent with a diagnosis of Lyme arthritis. A repeat MRI of the left knee on day 52 showed a large left knee joint effusion with synovitis and adjacent distal anterior thigh myositis without evidence of osteomyelitis or soft tissue abscess (Figure 2A). Orthopedics performed an irrigation and debridement on day 52, which revealed purulent loculation of joint fluid. The patient was treated with intravenous cefepime (50 mg/kg/dose every eight hours) for 48 hours for broader empiric coverage due to prior growth of an *Enterobacter* species. Synovial fluid and blood cultures were positive for *L. amnigena* by MALDI-TOF.

**Figure 2:**
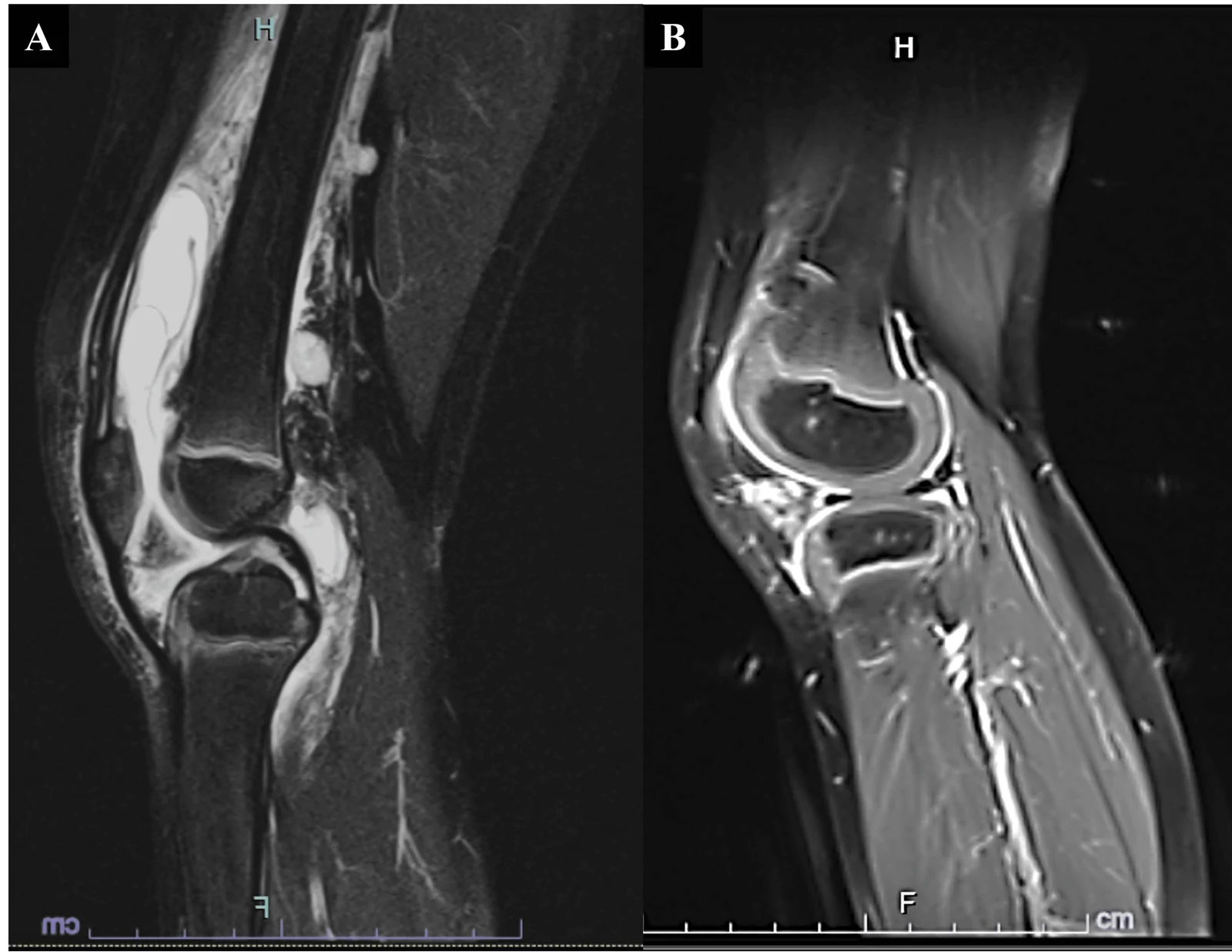
MRI of the left knee (A) Sagittal MRI of the left knee during her initial course showing joint effusion, uniform synovitis, and reactive popliteal lymph nodes. (B) Sagittal MRI of the left knee at the time of recurrence showing rim‑enhancing infrapatellar anteromedial fluid collection with surrounding inflammation. MRI: magnetic resonance imaging

She was discharged on day 54 with an improved knee exam and down-trending CRP to 2.4 mg/dL. She was ultimately transitioned to oral trimethoprim/sulfamethoxazole (TMP/SMX) at 6 mg/kg/day (TMP component) for two weeks. At the completion of this course, her CRP was normal, but ESR remained elevated at 57 mm/hr. The TMP/SMX dose was determined to be suboptimal, and she was increased to 11 mg/kg/day (TMP component) for an additional three weeks of therapy. The patient was lost to follow-up, and no additional laboratory evaluation was performed at the completion of therapy. Therefore, success of treatment, including full resolution of symptoms, reduction of pain scores, and increased activity, as well as adherence to pharmacologic treatment could not be assessed at that time.

Approximately 16 months after the completion of her prior antibiotic course (about 18 months after the onset of her initial infection), the patient presented to care with three weeks of left knee pain and swelling following a ground-level fall. Similar to her first injury, she had fallen on her left knee while playing in the grass and had no immediate pain or swelling, but this gradually developed after the fall. There was no evidence of a puncture wound or skin breakdown. At the repeat visit, the parent reported that the child's knee appeared to have fully recovered following her prior treatment with TMP/SMX, with no pain, swelling, or limited activity until the recurrence over the preceding three weeks. The patient had been healthy between episodes. On physical exam, she had a well-healed surgical scar on the left knee with swelling and tenderness to palpation of the anterior-medial aspect of the knee without erythema. The patient had no fever, rash, or other joint involvement.

Left knee MRI demonstrated a subcutaneous rim-enhancing fluid collection within the infrapatellar anteromedial knee, with significant surrounding inflammation (Figure 2B). She underwent two irrigation and debridement procedures (Figure 3), and cultures again grew *L. amnigena*, confirmed with MALDI-TOF. Due to this recurrence, she had a limited immune evaluation which was not suggestive of an underlying immunodeficiency.

**Figure 3:**
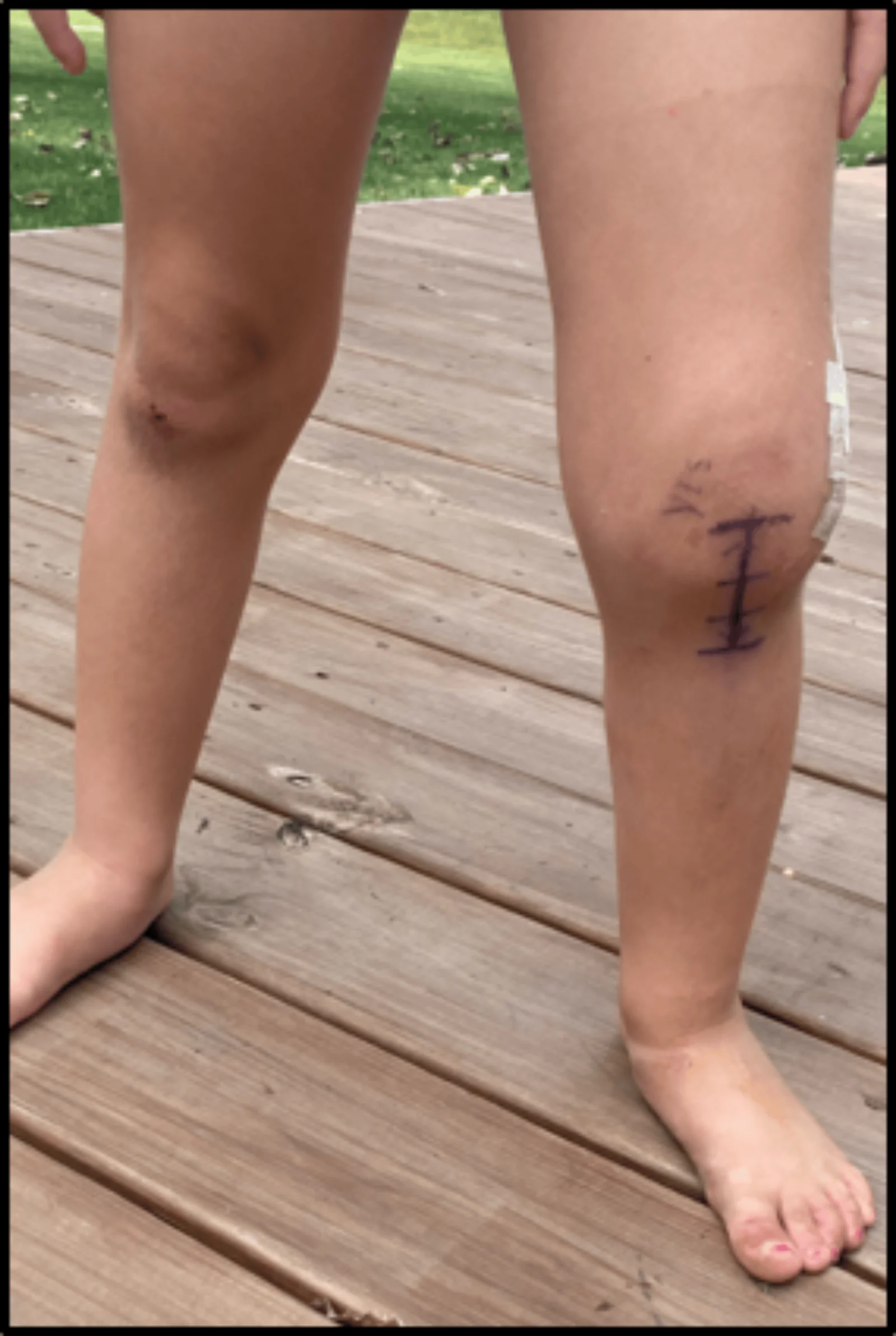
Left knee swelling The patient's knee following a repeat surgical procedure 18 months after her initial presentation due to recurrence of her infection.

The patient was treated with intravenous cefepime (50 mg/kg/day every eight hours) following the first surgery for five days. At discharge, she was switched to oral TMP/SMX (15 mg/kg/day TMP component divided every eight hours) for three months. The patient tolerated the antibiotic course well, with no reported clinical or laboratory adverse events. Her knee swelling completely resolved, and she was able to fully participate in all desired activities without limitation. She has remained free of recurrence for over two years at the time of this case report. Figure 4 showcases the timeline of the patient's care.

**Figure 4:**
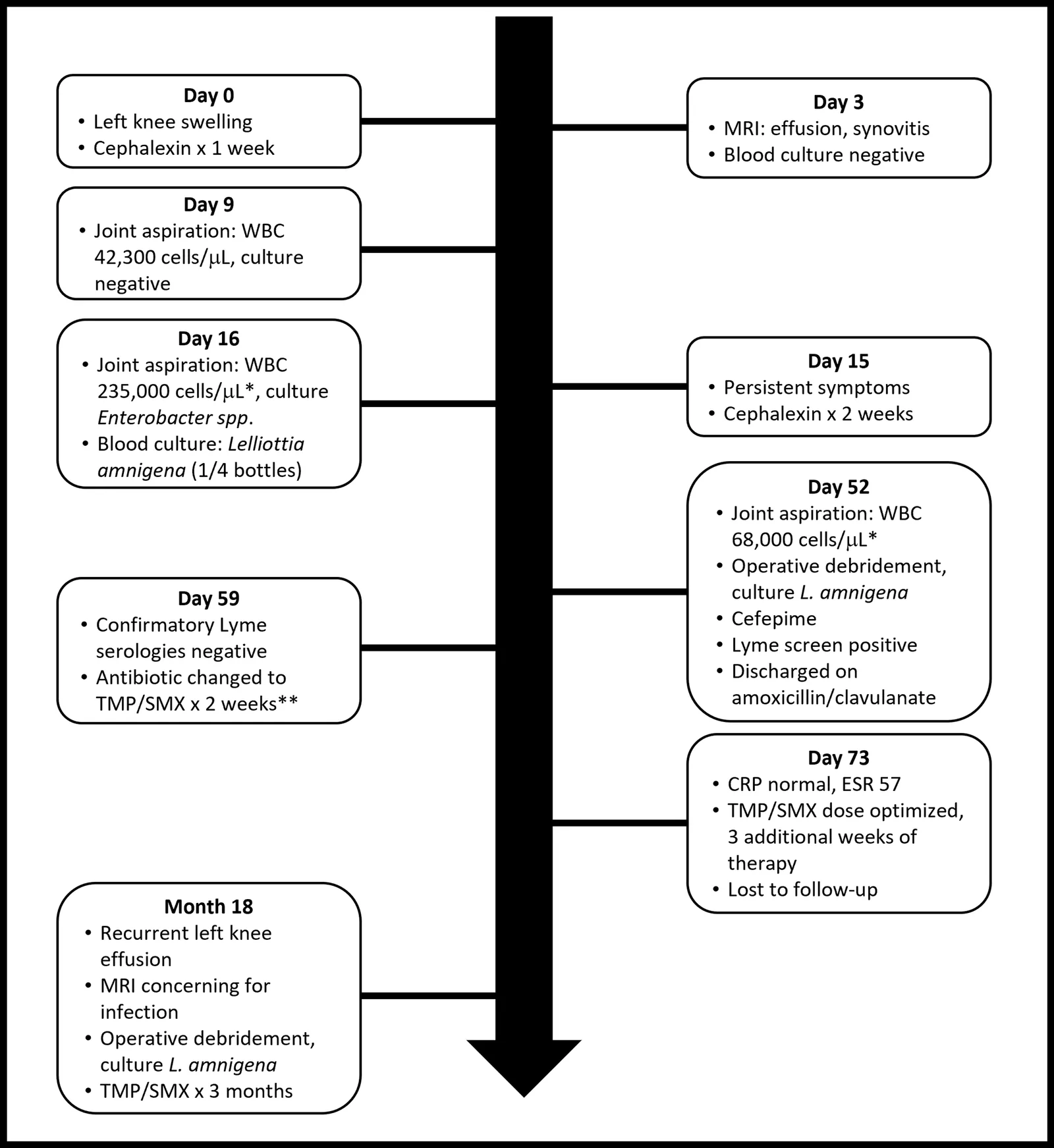
Clinical timeline Timeline (not to scale) of the patient's clinical course including antibiotics, imaging, and interventions beginning at the presentation of her first episode of septic arthritis and ending at the resolution of her second episode of septic arthritis. *>90% neutrophils **Drug susceptibility results: susceptible to aminoglycosides, tetracycline, TMP/SMX, ciprofloxacin, cefoxitin, cefepime; resistant to cefazolin by Kirby-Bauer disc diffusion MRI: magnetic resonance imaging; WBC: white blood cell count; TMP/SMX: trimethoprim/sulfamethoxazole; CRP: C-reactive protein; ESR: erythrocyte sedimentation rate

## Discussion

*L. amnigena* is a rare gram-negative rod in the *Enterobacteriaceae* family that is primarily known as a plant pathogen [3]. Human infections are uncommon and usually occur in the setting of risk factors such as immunosuppression [5,6], medical hardware [5], or following penetrating injuries [7,8]. Reported cases are sparse [4] and include bacteremia [9], urinary tract infections [10], peritonitis, and pneumonia [6].

To our knowledge, this is the first reported case of *L. amnigena* septic arthritis in a healthy child and the first to show recurrence after a long symptom-free period. The lack of known risk factors (immunodeficiency, hardware, or penetrating trauma) suggests the organism can cause invasive disease even in an otherwise healthy host. In our case, the patient had a minor fall preceding the injury, but it is uncertain if this was truly associated with her infection or if it was an example of recall bias; she had no known puncture wound or skin breakdown and was able to continue regular activities the day of the fall before developing swelling two days later.

Recurrent septic arthritis in children is rare, especially without structural joint problems or medical hardware. Possible explanations for our patient's recurrence include bacterial persistence in the joint or soft tissues with recrudescence 16 months after her initial treatment or reinfection from an environmental source. No foreign body was identified in any of the imaging tests or during operative intervention over the course of her infection that may have served as a nidus for recurrence. Bacterial persistence for months or years has been described for other organisms [11] and is favored in this case over reinfection given the extreme rarity of this organism causing infection in humans. *L. amnigena* can survive in diverse environments, but mechanisms of persistence in human tissue are not well understood. The initial culture isolates were not available for genomic comparison when the patient developed recurrence, preventing firm conclusions on the source of her recurrent infection.

The patient's diagnosis was not straightforward early in her course. The initial synovial fluid culture was negative prior to antibiotic therapy. When subsequent cultures were positive, inconsistent terminology between synovial fluid (*L. amnigena*) and blood cultures (*Enterobacter* spp.) impacted the clinical interpretation of the results, including the consideration that the isolates represented contaminants. In retrospect, the persistent symptoms with a positive blood culture for gram-negative bacteria, elevated inflammatory markers, and repeat positive synovial cultures supported true infection. Susceptibility testing guided TMP/SMX therapy, which correlated with clinical improvement.

There are no specific treatment guidelines for *L. amnigena* septic arthritis. Current guidelines for typical causes of acute hematogenous bacterial arthritis recommend 10-14 days of total therapy (intravenous and oral) [12]. Definitive source control with operative debridement did not occur until day 52 of illness, during which time bacterial persistence within the joint or soft tissues may have occurred. Notably, *L. amnigena* is capable of Amp-C induction, and first-generation cephalosporins such as cephalexin are unreliable against these organisms regardless of in vitro results. This likely contributed to her poor response to therapy early in her illness. While our patient received five weeks of therapy with TMP/SMX after source control, the dose was suboptimal for the first two weeks. In the setting of recurrence with an atypical gram-negative pathogen, the duration of antibiotic therapy should be individualized according to susceptibility results, the adequacy of source control, and the clinical response to treatment [5,10]. In this case, a three-month course after the second episode was successful. Close follow-up and collaboration with orthopedics and infectious diseases were important to the outcome.

## Conclusions

This case adds to the limited but growing body of literature on *L. amnigena* as a human pathogen. It shows that this organism can cause serious joint infection in healthy hosts and that recurrence or reinfection is possible even after initial treatment. When an unusual gram‑negative organism is isolated from a sterile site in the appropriate clinical context, it should not be dismissed as contamination. For rare causes of septic arthritis, the duration of antibiotic therapy should be individualized according to susceptibility results, the adequacy of source control, and the clinical response to treatment.
